# Disentangling Chemical Defence and Prey Composition of *Oophaga sylvatica*

**DOI:** 10.1007/s10886-026-01780-w

**Published:** 2026-11-10

**Authors:** Arianna Tartara, Karla Neira-Salamea, Alexander Keller, Mark-Oliver Rödel, Florian Frenzel, Michael Heethoff, Ralph A. Saporito

**Affiliations:** 1 Animal Evolutionary Ecology, Ecological Networks, Technical University of Darmstadt, Darmstadt, Germany; 2 Museum für Naturkunde - Leibniz Institute for Evolution and Biodiversity Science, Berlin, Germany; 3 Faculty of Life Sciences, Humboldt-Universität zu Berlin, Berlin, Germany; 4 Grupo de Investigación en Ecología y Evolución en los Trópicos-EETrop, Universidad de Las Américas, Quito, Ecuador; 5 Cellular and Organismic Networks, Faculty of Biology, Faculty of Biology, Ludwig-Maximilian-Universität, Münich, Germany; 6 Institute of Soil Science & Land Evaluation, Department of Soil Biology, University of Hohenheim, Stuttgart, Germany; 7 Department of Biology, John Carroll University, University Heights, Ohio, OH USA

**Keywords:** Alkaloids, Poison frogs, Ecuadorian Chocó, Predator-prey interactions, GC-MS, Metabarcoding

## Abstract

Poison frogs acquire alkaloids from arthropod prey, yet linking diet to chemical defence remains challenging due to methodological and ecological complexity. We investigated this relationship in 23 adult *Oophaga sylvatica* from an early-stage cacao regeneration plot in the Ecuadorian Chocó. Using non-destructive alkaloid sampling and GC-MS profiling, combined with stomach flushing, morphological prey identification, and DNA metabarcoding, we characterised individual alkaloid richness and abundance (total alkaloid quantity), described prey diversity, and tested whether diet similarity correlated with alkaloid similarity. We also compared metabarcoding and morphological identification for stomach content analysis.

We detected 130 alkaloids spanning 16 structural classes and found substantial inter-individual variation in richness and abundance. Most alkaloids occurred in low quantities, whereas a few classes dominated chemical profiles. Total alkaloid quantity increased with alkaloid richness. Diet analysis recovered 280 prey taxa across 22 orders, with ants, flies, beetles, and mites contributing most to richness. Metabarcoding recovered more diversity, improved taxonomic resolution, and detected highly digested and soft-bodied prey. Morphological identification recovered fewer taxa overall but performed better for small, sclerotized groups, particularly mites.

Prey composition could not be correlated with alkaloid profiles, likely reflecting temporal mismatches between short-term diet data and long-term toxin accumulation, limited knowledge of alkaloid sources, and methodological constraints. Using available literature, only 15% of detected alkaloids could be linked to known arthropod sources among the recovered prey. We demonstrate that combining morphological prey identification with DNA metabarcoding provides a more comprehensive understanding of poison frog diets while highlighting existing knowledge gaps regarding alkaloid sources and the need for more complete DNA barcode reference databases.

**Supplementary Information:**

The online version contains supplementary material available at https://doi.org/10.1007/s10886-026-01780-w.

## Introduction

Poison frogs are a well-known example of organisms with diet-derived chemical defences. Certain species in the families Dendrobatidae, Bufonidae, Eleutherodactylidae, Mantellidae and Myobatrachidae evolved the ability to sequester an array of lipophilic alkaloids from arthropod prey (Daly et al. 1994; Saporito et al. 2009). These aposematic anurans store alkaloids in cutaneous glands, resulting in diverse chemical profiles that mediate predator deterrence and may play roles in microbial defence (Saporito et al. 2012; Santos et al. 2016; Hovey et al. 2018). So far, studies have identified more than 1200 alkaloids belonging to 28 structural classes in the skin of poison frogs (Daly et al. 2005, 2008; Saporito et al. 2012; Garraffo et al. 2012; Hovey et al. 2018; Sague et al. 2024; R. Saporito unpublished data). The occurrence and abundance of specific alkaloids varies largely among species and between populations, and, to a lesser extent, also among individuals within populations, resulting in large-scale as well as small-scale heterogeneity in chemical defence profiles (Saporito et al. 2006, 2007a, b, 2010; Daly et al. 2007, 2008; Jeckel et al. 2015; McGugan et al. 2016; Basham et al. 2021).

Identifying the dietary sources of alkaloids in poison frogs is challenging, but several studies have provided direct evidence that particular arthropod lineages are sources of specific alkaloids (Saporito et al. 2007a, b, 2011, 2015; Jones et al. 2010; Fox and Adams 2022). Certain oribatid mites, several ant species, and one millipede species have been identified as dietary sources for alkaloids (Saporito et al. 2012; Heethoff et al. 2016; Jones et al. 2018), either through direct biosynthesis or by serving as carriers of these compounds. However, not all members within these taxonomic groups possess alkaloids. For instance, the widespread oribatid mite *Archegozetes* sp. is abundant in leaf litter and poison frogs diet (McGugan et al. 2016), but contains no alkaloids (Sakata and Norton 2003; Saporito et al. 2015). If prey are identified only to broad taxonomic levels (e.g. “Oribatida” or “Acari”), this could confound the identity of alkaloid-containing arthropod sources (Heethoff et al. 2016). Therefore, to avoid potentially masking important chemical-ecological patterns in frog-prey trophic interactions, it is crucial to achieve high taxonomic resolution in prey identification.

Methodologically, studies of poison frog diets have historically relied on morphological sorting and identification of stomach contents (e.g., Toft 1980; Caldwell 1996; Marín-Martínez et al. 2019). This approach allows direct observation of prey remains and is particularly effective for recovering intact specimens, and also provides quantitative information on prey size, number, and life stage. However, morphological identification is time-intensive, depends on the availability of sufficient taxonomic expertise, and is often limited when specimens are partially digested, fragmented, or lack diagnostic features, resulting in coarse taxonomic resolution.

In recent years, advanced molecular approaches such as DNA metabarcoding have emerged (Plewnia et al. 2026). Metabarcoding overcomes many limitations of morphological identification by detecting highly digested items and substantially increasing taxonomic resolution (Clare 2014). It is time-efficient, can be highly reliable, and provides standardized semi-quantitative data outputs (Egeter et al. 2015; Kopp et al. 2023). As a result, studies on non-dendrobatid anurans have increasingly adopted metabarcoding to achieve more comprehensive prey detection from stomach contents or fecal samples (Pereira et al. 2021; Dudczak et al. 2025).

Despite these advantages, metabarcoding also presents several methodological limitations. Successful taxonomic identification depends on the recovery and amplification of sufficiently preserved DNA, and PCR amplification can preferentially amplify abundant or high-quality DNA templates, potentially underrepresenting small or low-biomass taxa. In addition, accurate identification relies on the completeness of molecular reference databases (Porter et al. 2019; Holman et al. 2022). These challenges may be especially pronounced in tropical systems, where many arthropod taxa remain poorly represented in reference libraries (Basset et al. 2022), and where primer mismatches and incomplete databases can lead to false negatives or reduced taxonomic resolution (Elbrecht and Leese 2015; Deagle et al. 2019).

One important distinction between the two approaches is how digestion influences prey detectability. Morphological identification favours prey with structures that remain intact over longer periods, whereas metabarcoding depends on the persistence of amplifiable DNA and therefore tends to reflect more recently consumed prey (Amundsen and Sánchez-Hernández 2019). Therefore, both methods may be subject to distinct biases. In morphological datasets, differential digestion rates could lead to an overrepresentation of hard-bodied prey, as heavily sclerotized structures persist longer in the digestive tract while soft tissues are rapidly degraded. In contrast, metabarcoding biases may be influenced by prey size and biomass. Smaller organisms contain less tissue and could become undetectable more rapidly during digestion, while size-related DNA dilution in prey-rich stomachs may further reduce detection probability (Holman et al. 2022). This is particularly relevant in our system, where small, often sclerotized arthropods are of primary interest due to their potential role as alkaloid sources.

*Oophaga sylvatica* (Funkhouser 1956) is a dendrobatid poison frog that occupies humid lowland and foothill habitats in the north-western Andes and the Chocó region (Ron et al. 2024). This species exhibits substantial variation in alkaloid richness and abundance, even among co-occurring sympatric individuals, potentially reflecting lifetime accumulation, individual selectivity, microhabitat differences or, simply, stochastic prey encounters (McGugan et al. 2016; Moskowitz et al. 2020; Tarvin et al. 2024; Martin et al. 2025). This variability makes *O. sylvatica* an ideal case study for comparing traditional prey analysis with modern metabarcoding approaches. To our knowledge, DNA metabarcoding-based prey identification has not yet been systematically applied to chemically defended anurans. In contrast, current knowledge on *Oophaga sylvatica* diet relies mainly on morphological prey identification (Tarvin et al. 2024; Martin et al. 2025), with a few studies combining this approach with DNA barcoding of selected prey taxa (McGugan et al. 2016; Moskowitz et al. 2020).

**Fig. 1:**
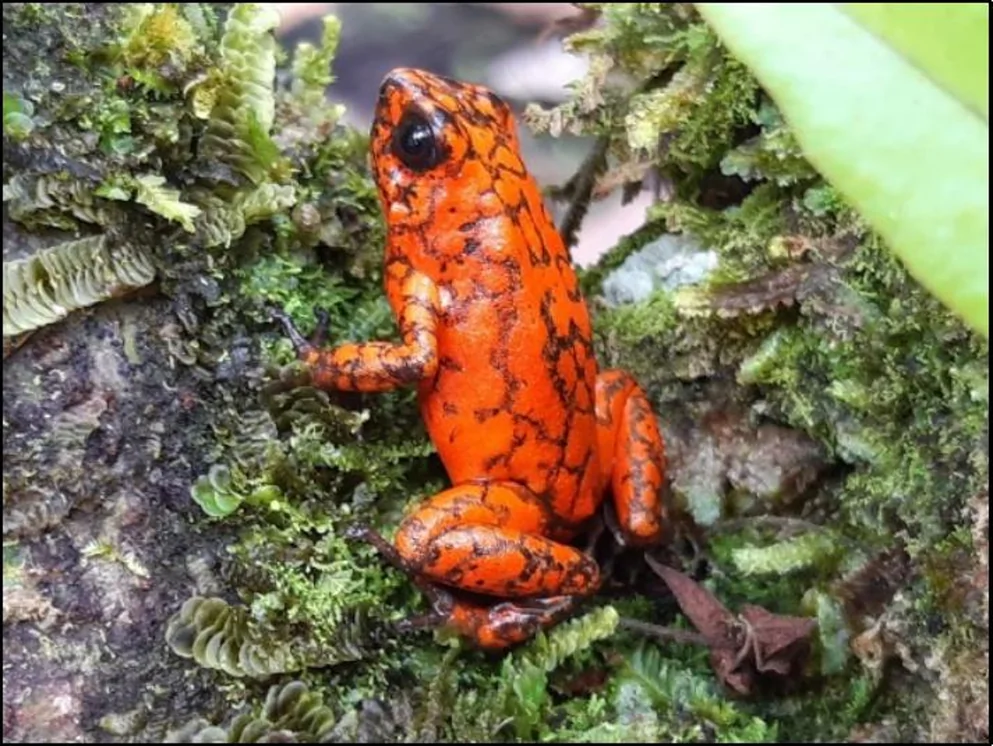
Adult *Oophaga sylvatica* on a *Theobroma cacao* tree; photograph courtesy of M. Rödel

In our study, we coupled non-destructive alkaloid sampling and GC-MS profiling with stomach flushing, morphological prey identification, and DNA metabarcoding. We applied this integrative approach to analyse a chemically defended trophic system in 23 adult *O. sylvatica* sampled from an early-stage cacao regeneration plot in the lowland Ecuadorian Chocó. Our objectives were to (1) characterise individual-level alkaloid richness, abundance, and structural class composition in *O. sylvatica*; (2) describe individual-level prey diversity and composition while evaluating the relative performance of metabarcoding and morphological identification for diet reconstruction in poison frog trophic systems; and (3) test whether diet composition correlates with alkaloid similarity. We hypothesised that (1) alkaloid and diet profiles exhibit high intraspecific variation; (2) morphological and metabarcoding approaches detect partially distinct prey spectra due to their different methodological biases and temporal resolutions, making their combined use superior for comprehensive reconstruction of chemically relevant diets; and (3) dietary and alkaloid composition covary among individuals. Because only a subset of consumed prey are potential alkaloid sources, we additionally assessed the recovered prey taxa for their documented association with alkaloid sequestration through a literature-based review.

## Methods and Materials

### Field Sampling

Sampling was conducted within the `Reassembly` Research Unit (www.reassembly.de), which investigates tropical forest recovery in the lowland Ecuadorian Chocó (Esmeraldas Province). For the present study, we selected an early-stage forest regeneration site (plot CR13 in the `Reassembly` design) whose detailed characteristics are provided in Escobar et al. (2025). The study site consists of a former cacao (*Theobroma cacao*) plantation of 2500 m^2^, abandoned one year prior to the start of our study. We investigated 23 adult *Oophaga sylvatica* between April 2022 and May 2023, as part of a wider campaign aimed at monitoring the whole leaf-litter anuran community (Neira-Salamea et al. 2026). All *O. sylvatica* were captured by hand and placed in individual plastic bags for subsequent investigations.

We used a transcutaneous amphibian stimulator (TAS) for non-destructive skin alkaloid extraction (Grant and Land 2002), and stomach flushing to obtain non-destructive diet samples. TAS parameters were standardised for all individuals (frequency: 50 Hz; pulse width: 2 ms; amplitude: 9 V). Following stimulation, we collected skin secretions by wiping the frog with absorbent bibulous paper disks, which were immediately placed into 1 ml of 100% methanol in glass vials with Teflon-lined caps (Bolton et al. 2017; Basham et al. 2021; Porras-Brenes et al. 2025). TAS provides a standardized, non-destructive estimate of alkaloid quantity recovered under the specified procedure, but does not measure the total alkaloid content of the frog’s skin. Therefore, the alkaloid quantities reported here do not represent the total quantity of skin alkaloid present in *O. sylvatica*, and should be compared based on their relative differences. We express alkaloid quantities as µg per frog. We allowed 5 min for complete recovery from stimulation, and then proceeded with diet collection through stomach flushing (Solé et al. 2005; Schäfer et al. 2022). We used a soft plastic catheter of ca. 2 mm diameter attached to a 20 ml syringe filled with rainwater which was inserted via the oesophagus into the stomach. Regurgitated stomach contents were collected in plastic sieves with tea filter paper and preserved in 75% ethanol for subsequent analyses. For each individual, sampling date was recorded (Table S1). Each frog was treated as an independent sampling unit. Body traits (snout-vent length, head width) were measured (Table S1), and all frogs were photographed in order to be able to identify individuals. Frogs were released at their capture locations immediately following recovery. All procedures adhered to ethical guidelines for amphibian research compiled by the American Society of Ichthyologists and Herpetologists (ASIH), The Herpetologists’ League (HL) and the Society for the Study of Amphibians and Reptiles (SSAR), and were conducted under the relevant Ecuadorian collection and research permits.

### Alkaloid Analysis

Methanolic skin extracts were spiked with 10 µg of nicotine (≥ 99%, Sigma-Aldrich, Milwaukee, WI, USA) as an internal standard. Nicotine was chosen because its retention time (Rt) lies outside the range for most alkaloids, and has been used in other studies of poison frogs (e.g. Bolton et al. 2017; Basham et al. 2021; Porras-Brenes et al. 2025). Extracts were evaporated to dryness under nitrogen and reconstituted in 500 µl of 100% methanol. Extracts were stored at -20 °C until analysis. We analysed alkaloids using a Varian Saturn 2100T ion trap mass spectrometer coupled to a Varian 3900 gas chromatograph equipped with a 30 m × 0.25 mm i.d. Varian Factor Four VF-5 ms fused silica column. The GC was run in split mode (20:1), the injection port temperature was 250 °C, and sample injection volume was 1 µl. The GC temperature program ran from 100 °C to 280 °C at 10 °C min-1, with an initial solvent delay of four minutes, and helium as the carrier gas (1 ml min-1). Each sample was run in triplicate under electron impact (EI) ionisation (70 ev), and once using chemical ionisation (CI) with methanol as the reagent gas (CI storage level: 19 m/z; Ejection Amplitude: 15 v; Background mass: 55 m/z). Mass spectra were recorded in full scan mode (50–400 m/z). Sample order within and across batches was randomised to minimise potential run-order effects.

Instrument blanks consisting of 100% methanol were analysed at the beginning of each batch to check instrument cleanliness and detect residual compounds from previous analytical batches. No procedural extraction blanks or TAS sampling blanks were included. Therefore, carryover from one sample to the next within a batch was not assessed directly. The GC-MS system was cleaned daily, and the injector liner and septum were replaced regularly following manufacturer recommendations and instrument performance checks. We identified alkaloids by comparing retention times of chromatographic peaks and mass spectral fragmentation patterns to published reference data for poison frog alkaloids (Daly et al. 2005; Saporito et al. 2006; Bolton et al. 2017; Hovey et al. 2018; R. Saporito unpublished data). Retention indices were not used. Quantification was performed relative to the nicotine internal standard based on the integrated peak area from the total ion current (TIC) chromatograms using a Varian MS Workstation v.6.9 SPI. For each alkaloid, values from the three replicate EI injections were averaged. No predefined acceptance criteria or precision threshold were applied to assess agreement among replicate injections. The limit of quantification (LOQ) was 0.1 µg; alkaloids below this threshold were treated as absent and were not included in subsequent analyses. Alkaloids are reported using the established code system, where a bold number indicates nominal molecular mass and a bold letter distinguishes alkaloids with the same mass (Daly et al. 2005).

### Prey Identification

#### Morphological Analysis

Stomach contents were examined under a stereomicroscope, and prey items were first classified to arthropod orders. We then refined these identifications to the morphospecies level using order- and region- specific taxonomic keys, drawing on published literature (references in Annex 1) as well as a reference collection of previously barcoded arthropods gathered in situ during a leaf litter prey-availability pilot study (A. Tartara unpublished data). All corresponding barcode sequences are publicly available in BOLD (project: ARTA, https://portal.boldsystems.org).

Morphospecies were defined as operational taxonomic units distinguished based on consistent and diagnosable morphological characters when formal species-level identification was not possible (Oliver and Beattie 1996; Krell 2004). During identification, items were photographed and preserved in molecular-grade 98% ethanol for subsequent DNA analysis by metabarcoding. Body length and width for each undigested prey item were measured in ImageJ^®^, and prey volume was estimated using the prolate spheroid formula (Valderrama Vernaza et al. 2009):

1 $$\:V=\:\frac{4}{3}\pi\:\left(\frac{length}{2}\right){\left(\frac{width}{2}\right)}^{2}$$

#### Metabarcoding Analysis

Arthropod mixed samples were extracted using the Qiagen DNeasy^®^ 96 Blood & Tissue Kit and processed for Illumina-based COI metabarcoding using the minibarcode primers mlCOIintF (GGWACWGGWTGAACWGTWTAYCCYCC) and HCO2198 (TAAACTTCAGGGTGACCAAAAAATCA), as described in Leray et al. (2013). Each biological sample was amplified in three PCR replicates. Six negative controls, comprising extraction and blank PCR negative controls (NC and BPCR), and four positive controls (PC) containing *Bombus terrestris* were included to monitor contamination and sequencing artefacts. Libraries were prepared using a two-step PCR protocol with dual indexing. After PCR, DNA amounts were normalized among samples using SequalPrep Normalization Plate Kits (Applied Biosystems), quality controlled, and sequenced alongside other samples on an Illumina NovaSeq X using paired-end 2 × 300 bp sequencing at AllGenetics (Spain). Raw sequence reads were merged, quality filtered (ee < 1.0), dereplicated, denoised, and checked for chimaeras to generate amplicon sequence variants (ASVs) using an established bioinformatic workflow (https://github.com/chiras/metabarcoding_pipeline, Leonhardt et al. 2022) and VSEARCH (Rognes et al. 2016).

Among biological samples remaining after sequence processing and quality filtering, the median raw read count was 32,328 (range: 10,826–144,877), and the median final read count after filtering was 28,690 (range: 10,181–128,823). Samples with fewer than 1,000 reads after filtering were excluded from downstream analyses. Sequencing depth in biological samples and negative controls is illustrated in Figure S1.

Quality-control assessments of negative and positive controls were performed across the entire sequencing project, including samples not analysed in the present study, because these samples were processed and sequenced as part of the same `Reassembly` Research Unit. The control results were therefore used to assess potential contamination and sequencing artefacts at the project level.

Negative controls were evaluated by comparing their read counts and taxonomic composition with those of biological samples. Final read counts in negative controls ranged from 18 to 503. To distinguish background detections from potential external laboratory contamination, the log₂ enrichment of taxa in negative controls relative to biological samples was assessed (Figure S2). An empirical bulk mean was estimated by iterative trimming to characterise the background distribution of enrichment, and taxa exceeding the outlier threshold (bulk mean + 2 SD) were considered anomalously enriched. Six taxa exceeded this threshold and were removed from downstream analyses. This approach distinguished taxa disproportionately enriched in negative controls from those occurring at levels consistent with the background distribution across the sequencing project.

Positive controls containing *Bombus terrestris* were used to assess index-jumping and cross-contamination among samples. The relative abundance of positive-control-associated taxa detected in non-positive-control samples was examined to quantify potential signal spillover. Based on the observed spillover, a per-sample relative-abundance threshold of 0.1% (relative abundance < 0.001 on a 0–1 scale) was applied to remove low-frequency detections potentially attributable to index misassignment, tag jumping, or cross-contamination (Figure S3).

ASVs were postclustered at 97% sequence identity to generate pASVs and subsequently taxonomically assigned using VSEARCH global alignments (best hit, ≥ 97% identity) against an arthropod reference database originally downloaded from the BOLD DNA Barcode Reference Library on 27 June 2025 (https://www.boldsystems.org/data/data-packages), and subsequently curated using the scripts available at https://github.com/chiras/database-curation (Quaresma et al. 2024). Taxa without a direct match were searched against the same database using two complementary approaches to infer higher-level taxonomy: (1) BLAST local alignments followed by least common ancestor (LCA) assignment based on the first 100 hits with ≥ 90% identity and ≥ 90% coverage, and (2) SINTAX (Edgar 2016) implemented in VSEARCH. Results from both approaches were combined, and congruent taxonomic ranks were assigned to the deepest supported level. pASVs not assigned at least to class level were considered artefacts and removed, together with vertebrate sequences, including host and human contamination. The resulting pASVs were retained as individual molecular taxonomic units and assigned either to a formally identified species where species-level identification was supported, to higher taxonomic levels such as genus, family, or order, or to a Barcode Index Number (BIN). Raw sequencing reads of the whole project are deposited in the NCBI Sequence Read Archive under BioProject PRJNA1523123 (http://www.ncbi.nlm.nih.gov/bioproject/1523123). The analysis code, per-sample raw and filtered read counts, and associated metadata are available on Figshare at https://doi.org/10.6084/m9.figshare.32743443.

### Statistical Analysis

All statistical analyses were conducted in R v.4.3.1 (R Core Team 2023). Statistical significance was evaluated at *P* < 0.05 for all analyses.

To assess how completely our sampling captured the alkaloid diversity present in the studied *O. sylvatica* population, we estimated sample coverage and asymptotic richness using the iNEXT framework (Chao et al. 2014, 2023; Hsieh et al. 2016). We treated each alkaloid as an incidence-based “species” and used the number of frogs in which it occurred as incidence frequency data. Rarefaction and extrapolation were computed using Hill numbers and Chao2 estimators to account for the influence of rare compounds (alkaloids detected in only one or two frogs). We generated curves of richness and sample coverage as a function of sample size, and extracted asymptotic estimates for alkaloid richness, Shannon diversity, and Simpson diversity. Sampling completeness was calculated as the ratio of observed to estimated diversity.

To characterize how alkaloid defences were distributed within the population, we first examined the distribution of mean individual alkaloid abundances across all detected compounds. Because this distribution was strongly right-skewed (Figure S4), indicating that most alkaloids occur at low abundance while few dominate the overall chemical profile, we followed up by testing whether individuals possessing a richer alkaloid repertoire also accumulated a higher total alkaloid quantity. For each frog, we fitted a linear model with total alkaloid quantity (µg) as the response and alkaloid richness as the predictor. Model assumptions were verified visually and with a Shapiro-Wilk test on the residuals. Secondly, we generated binary presence-absence matrices at both the individual compound level and the structural class level as well as matrices based on relative abundances. Dissimilarity among individuals was calculated using the Jaccard index for binary data (presence-absence) and Bray-Curtis distances for abundance data, implemented in the *vegan* package (Oksanen et al. 2001).

To characterize how diet profiles were distributed within the population, we first combined morphological and metabarcoding identifications based on a hierarchical matching procedure (Table S2). For each frog, morphological and metabarcoding records were compared sequentially at the species, genus, and family levels, with agreement in higher taxonomic ranks required for each match. Species-level matches were identified first, followed by genus-level and then family-level matches among records not already matched at a higher taxonomic resolution. No matches required resolution at the order level. Within each frog, duplicate records sharing the same combination of order, family, genus, and species were collapsed to a single record. A concordant record was defined as a morphological and metabarcoding detection sharing a taxonomic assignment at the same hierarchical level, and contributed a single integrated record using the metabarcoding taxonomic assignment. Records that could not be reconciled at any of these levels were retained as method-specific records. Throughout analyses combining morphological and metabarcoding data, we use the term “prey taxon” to encompass formally identified species, morphospecies, and individual molecular taxonomic units.

The resulting integrated dataset was used to generate binary presence–absence matrices for prey taxa for each frog. We then calculated dissimilarity among individuals using the Jaccard index (*vegan*).

To evaluate the two prey identification methods, we compared metabarcoding and morphological identification using two complementary analyses based on paired samples, defined as frogs for which prey observations were available from both methods. Of the 23 frogs, 19 met the criteria for paired analysis, with prey observations available from both methods; four had metabarcoding observations only.

First, we examined differences in the prey community recovered by each method using alpha diversity (taxon richness per sample) and beta diversity (community composition based on Jaccard dissimilarity). Taxon richness was calculated for each frog and method as the number of distinct taxonomic assignments. Differences in alpha diversity were tested using paired Wilcoxon signed-rank tests. Effect sizes were reported as rank-biserial correlations, and the Hodges–Lehmann estimator was used to estimate the paired difference and its 95% confidence interval. Differences in beta diversity were assessed using PERMANOVA (adonis2: mat ~ Method; Jaccard dissimilarities). Permutations were restricted within frog identity, allowing method labels to be exchanged only within pairs. For the 19 paired frogs, 524,288 possible paired permutations were evaluated. Differences in multivariate dispersion between methods were assessed using PERMDISP (betadisper followed by permutest) with the same restricted permutation structure.

Second, we evaluated whether prey size influenced detection probability. We focused on the subset of morphologically identified taxa, including both overlap taxa (detected by both methods) and morphology-only taxa. Prey volumes were then compared between items detected by metabarcoding and those detected exclusively through morphological inspection using a Wilcoxon rank-sum test. We additionally performed a bootstrap analysis (10,000 iterations) to estimate the 95% confidence interval of the median difference.

To assess potential effects of frog traits and sampling period on alkaloids profiles and prey composition, we fitted multivariate models relating snout-vent length (SVL) and head width (HW) to the corresponding dissimilarity matrices. Analyses were conducted using Jaccard distances for presence-absence data and Bray-Curtis distances for relative abundance data. Permutations were restricted within sampling events to account for the non-independence of frogs sampled on the same date. Significance was assessed using PERMANOVA (adonis2, *vegan*) with 999 restricted permutations. All samples were included except one with a missing body measurement (*N* = 22).

Finally, to test whether similarity in diet composition corresponded to similarity in alkaloid profiles, we conducted Mantel tests (Pearson’s correlation, 999 permutations) on Jaccard dissimilarity matrices derived from the binary prey and alkaloid datasets. Similarity was assessed at both the alkaloid compound and structural class levels. For each analysis, we calculated Mantel *r* values and permutation-based P values.

### Literature-Based Alkaloid Source Assignment

To identify potential dietary sources of alkaloids detected in *Oophaga sylvatica*, we conducted a targeted review of the literature on alkaloid-bearing arthropods associated with poison frog chemical defence. Searches were performed in Web of Science and Google Scholar using combinations of terms related to poison frog alkaloids, arthropod-derived alkaloids, known alkaloid-producing taxa, and specific alkaloid classes.

We screened studies reporting alkaloid occurrence in arthropods and extracted information on both alkaloid identity and the taxonomic identity of the source organism at the species, genus, or family level. Literature-derived records were subsequently compared with prey taxa recovered from stomach contents to identify potential matches between detected alkaloids and candidate dietary sources.

For each frog, an alkaloid was assigned a possible source only when it co-occurred with a prey taxon reported in the literature to contain that compound. Because prey identification was not always resolved to species level, matches were evaluated at the highest shared taxonomic resolution available.

## Results

### Alkaloid Profiles

We detected a total of 130 distinct alkaloids across the sampled *O. sylvatica* population. Of these, 111 compounds could be classified into 16 established structural classes, whereas the remaining 19 could not be assigned to a specific structural class and were therefore categorized as ‘unclassified’ (abbr. ‘Unclass’). Among the 130 alkaloids, 10 compounds could not be matched to previously published mass spectral data, but their fragmentation patterns and chromatographic behaviour confirmed that they are likely novel poison frog alkaloids (Table S3). We observed high intraspecific variation in both richness and relative quantity (abundance) of alkaloids. Individual frogs contained between 6 and 61 alkaloids (median = 45; IQR = 16.5), and total alkaloid quantity per specimen ranged from 1.1 to 234.0 µg (median = 66.1 µg; IQR = 103.0).

Rarefaction and extrapolation curves indicated that richness increased with sample size and had not yet reached an asymptote (Figure S5). The Chao2 estimator predicted ca. 152 alkaloids in the population, compared to the 130 detected, corresponding to 86% estimated sampling completeness. However, diversity estimates based on Shannon and Simpson indices were higher (ca. 94% and 97%, respectively), indicating that the remaining undetected compounds consist of predominantly rare alkaloids (Table S4). Our sampling captured a substantial portion of the alkaloid diversity, showing evidence of modest but acceptable saturation. High completeness for abundant alkaloids is especially important to support the robustness of our following downstream analysis, hence their respective multivariate and ecological interpretations.

Alkaloid occurrence varied markedly among compounds. Only ca. 30% of alkaloids were present in more than half of the individuals, whereas ca. 40% of alkaloids occurred less frequently within our population (in less than 20% of frogs). The most widespread alkaloids were the tricyclic **193B**, 3,5-disubstituted pyrrolizidine **251 K**, 2,5-disubstituted decahydroquinoline *trans*-**269AB**, and 2,5-disubstituted pyrrolidine **197B**, each detected in 87–96% of frogs. In contrast, ca. 20% of alkaloids were found in only one specimen.

The patterns of relative alkaloid quantity were similarly uneven. The mean quantity of individual alkaloids across frogs ranged from 46.7 µg to our limit of quantification (LoQ = 0.1 µg). The distribution of mean individual alkaloid abundances was strongly right-skewed, with most alkaloids occurring at low quantities and a few contributing disproportionately to the overall chemical profile (Figure S4). The most abundant alkaloids were the histrionicotoxin **291 A**, Unclass 269 (Rt 15.78), histrionicotoxin **285 A**, and decahydroquinoline *trans*-**269AB**, which together constituted 45% of the total detected alkaloid quantity.

Frogs with a more diverse composition of skin toxins also carried substantially higher amounts of alkaloids (Figure S6). Across individuals, total alkaloid quantity increased significantly with alkaloid richness (estimate = 2.8, 95% CI = 1.11–4.54, *F*_1,21_ = 11.68, *P* = 0.003), explaining 36% of the variation (*R*^*2*^ = 0.36).

**Fig. 2:**
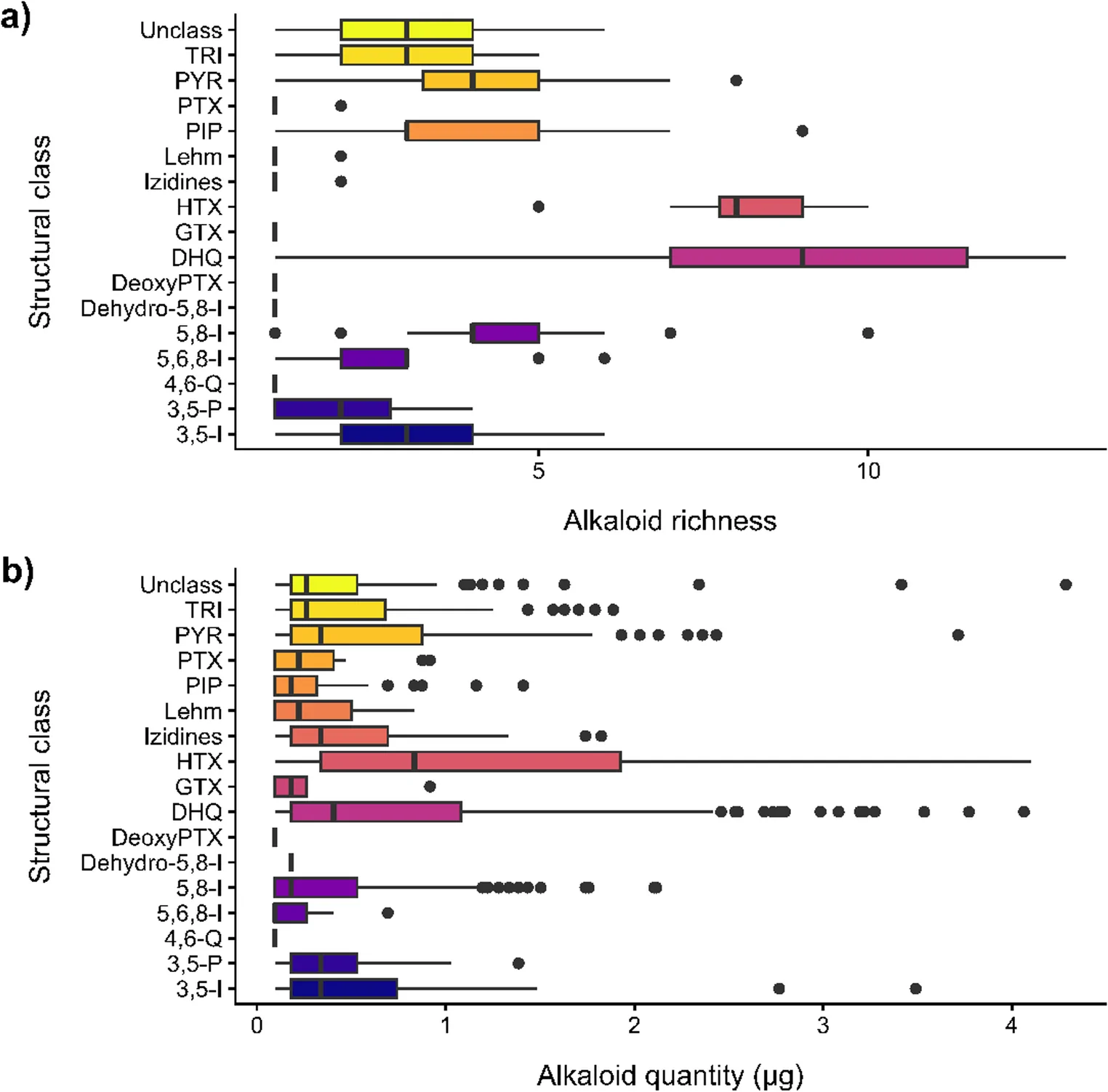
Structural class richness and quantity of skin alkaloids in the sampled *Oophaga sylvatica* population (*N* = 23 individuals); alkaloids are grouped into established structural classes (e.g., PTX, HTX, DHQ); boxplots show the median, interquartile range, and whiskers extending to the most extreme observations within 1.5 × IQR; points beyond the whiskers represent individual observations classified as outliers; Panel (**a**) displays alkaloid richness, expressed as the number of unique compounds detected within each structural class for each frog. Panel (**b**) shows the quantity of individual alkaloids within each structural class, with quantities log(1 + µg) transformed to facilitate comparison among classes with highly skewed distributions

Structural classes differed in both frequency and abundance (Figure S7). 2,5-Disubstituted decahydroquinolines (DHQ) and histrionicotoxins (HTX) were the most diverse (18 and 12 unique alkaloids, respectively) and collectively contributed the largest proportion of total alkaloid mass (DHQ: 617.0 µg; HTX: 832.0 µg), whereas pumiliotoxins (PTX, deoxyPTX), 4,6-disubstituted quinolizidines (4,6-Q), dehydro-5,8-indolizidines (dehydro-5,8-I), and a gephyrotoxin (GTX) occurred infrequently (only 1 or 2 individuals) and at low concentrations (total alkaloid mass of 0.1–6.30 µg). Chemical composition differed substantially among individuals, with frogs varying in both richness and relative quantity of alkaloids (Fig. 2). However, the population exhibited a consistent chemical signature dominated by a small set of widespread and abundant alkaloids.

### Diet Composition

The analysis of diet composition revealed a diverse prey community consumed by the different *O. sylvatica* individuals (Figure S8). Across all analysed prey items, we identified 280 unique prey taxa, belonging to 88 families and 22 orders, respectively. Consumed prey taxon richness varied substantially among frogs, with individuals consuming on average 22.7 ± 9.1 taxa (median = 24, range = 7–39) belonging to 6.4 ± 1.7 orders (median = 6, range = 4–12).

**Fig. 3:**
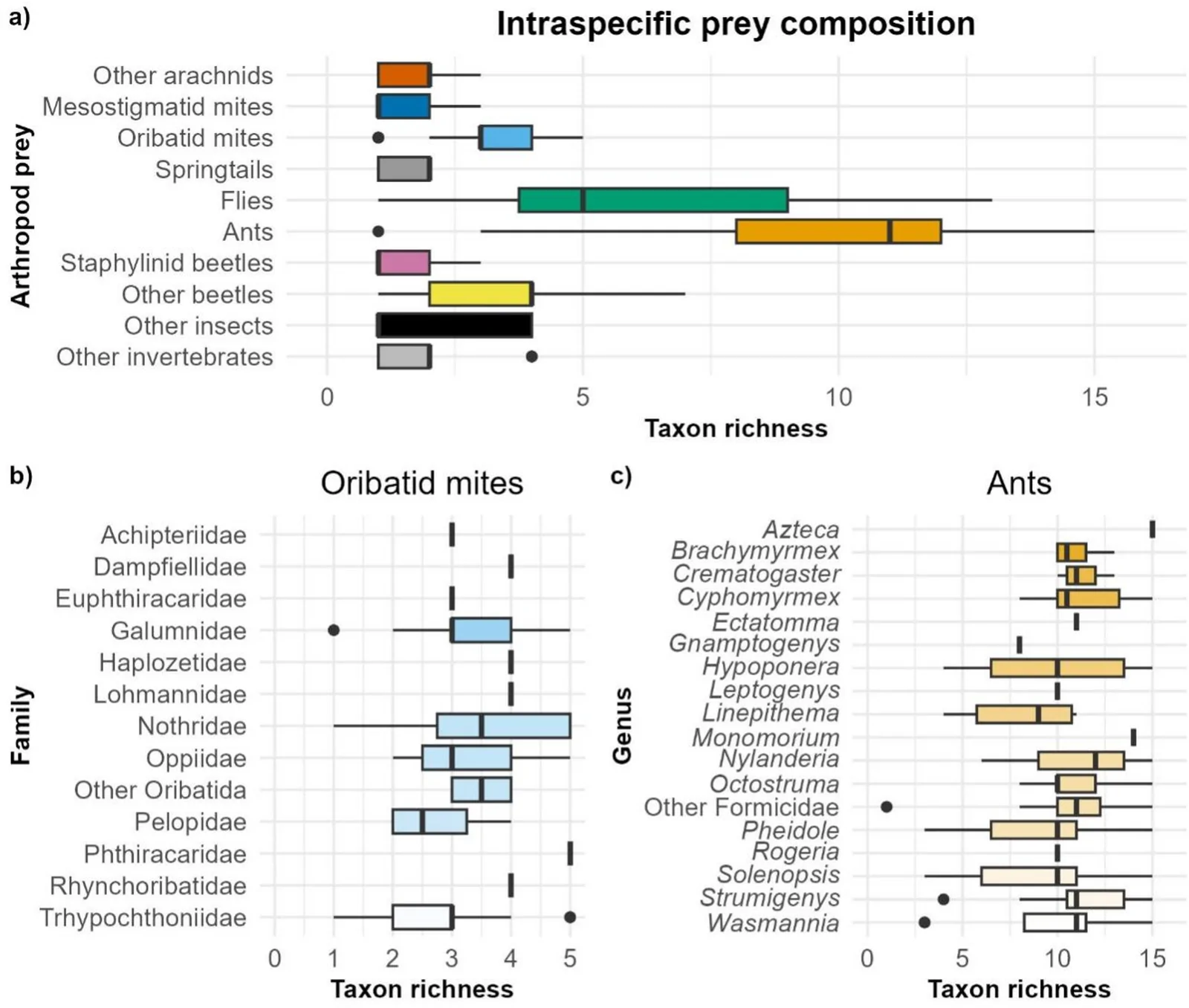
Intraspecific variation in prey composition and taxonomic richness in the sampled *Oophaga sylvatica* population (*N* = 23 individuals); boxplots show medians and interquartile ranges, with whiskers extending to the most extreme observations within 1.5 × IQR; points beyond the whiskers represent individual observations classified as outliers. Panel (**a**) displays prey richness across broad taxonomic categories, expressed as the number of prey taxa detected per frog within each group (e.g., ants, mites, beetles). Panel (**b**) shows prey taxon richness within oribatid mite prey (Oribatida), resolved to family level. Panel (**c**) shows prey taxon richness within ant prey (Formicidae), resolved to genus level

Our results indicate that although frogs consumed a broad range of arthropods, diet richness was dominated by ants, flies, beetles, and mites, with a subset of taxa occurring in high frequency across individuals (Fig. 3). Hymenoptera, Diptera, and Coleoptera were the most taxon-rich insect orders, comprising 81, 76, and 39 prey taxa, respectively, followed by the arachnid taxa Oribatida (32 prey taxa) and Mesostigmata (16 prey taxa). Consequently, Hymenoptera, Diptera, Oribatida, and Mesostigmata appeared in almost all stomach samples, whereas Coleoptera was present in only 15 frog stomachs. Among the identified prey, several taxa were recurrently detected across individuals. The oribatid mite *Archegozetes* sp. (BIN: ACQ7690) was the most frequent prey item, occurring in 17 frogs. The ant *Solenopsis terricola* occurred in 15 individuals, and *Pheidole zeteki* in 13 frogs. Other frequently recorded taxa included *Pheidole flavens*, an unidentified taxon in Mesostigmata (Parasitidae spp.), Nothridae sp. (BIN: AGB1955), and *Octostruma* sp. (BIN: ADH2162). Several ant taxa (*Pheidole* sp. (BIN: ACZ5524), *Wasmannia auropunctata*,* Solenopsis sp.* (BIN: ACY3042 and ADG8264)) and springtails (Paronellidae spp.) also appeared in 7–8 frogs.

### Prey Identification Methods Assessment

**Fig. 4:**
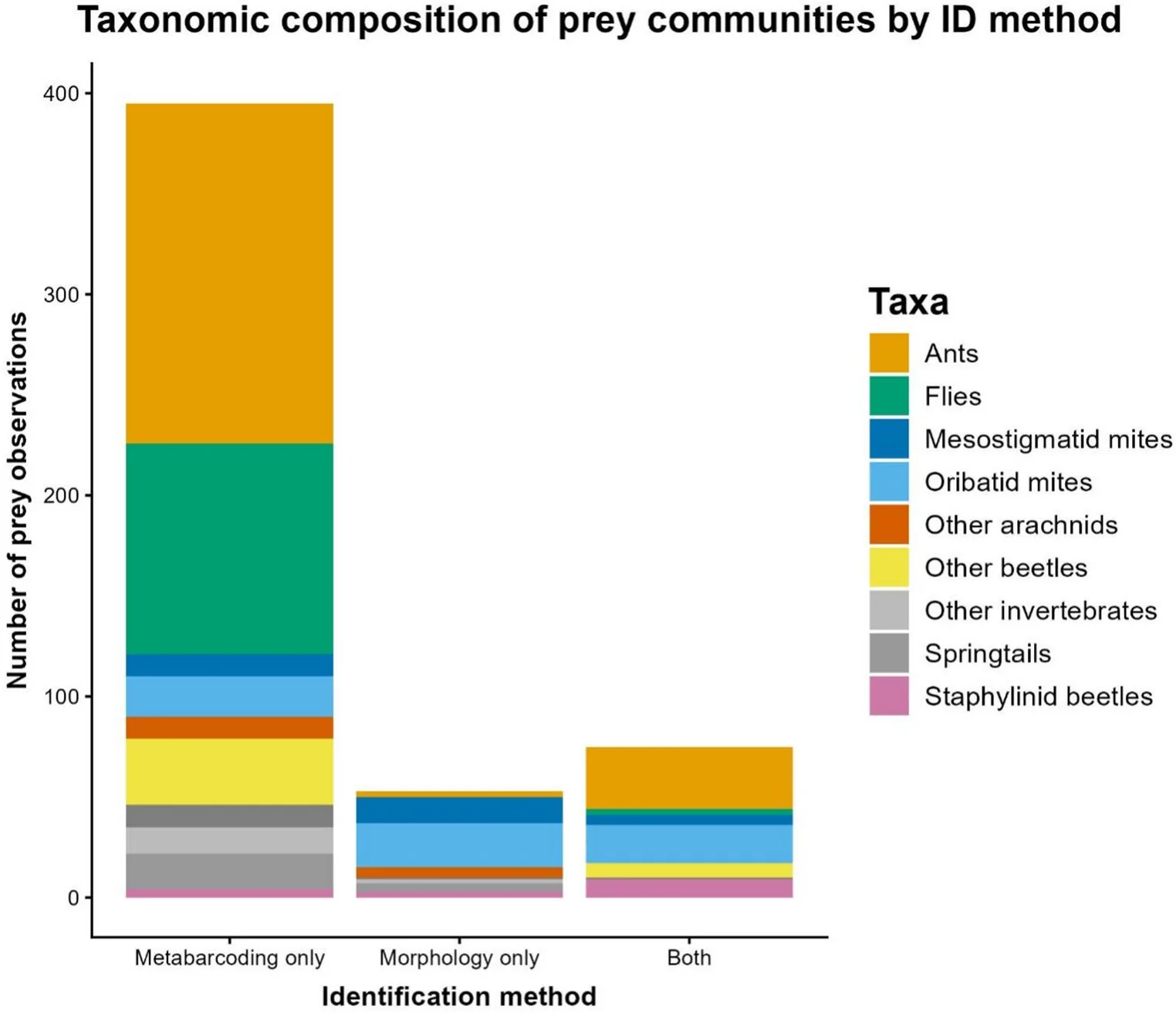
Taxonomic distribution of the 523 prey observations identified by metabarcoding and morphological identification; stacked bars represent the number of prey observations detected exclusively by metabarcoding (‘Metabarcoding only’), exclusively by morphological identification (‘Morphology only’), or by both methods (‘Both’); prey observations are grouped into broad taxonomic categories to facilitate comparison between identification methods

Morphological identification and metabarcoding differed substantially in the number and distribution of prey records recovered (Fig. 4). Across the complete combined dataset, 523 prey records were retained. Of these, 395 records were detected exclusively by metabarcoding, 53 exclusively by morphological identification, and 75 by both methods.

Across paired samples (*N* = 19), metabarcoding detected significantly higher taxon richness per frog than morphological identification (paired Wilcoxon signed-rank: *V* = 190, *P* < 0.001; effect size *r* = 0.878, Hodges–Lehmann estimate = 17 taxa, 95% CI = 12.5–22). Median richness was 20 taxa per frog for metabarcoding compared with 2 taxa for morphological identification (means = 20.2 and 2.79 records, respectively). Community composition did not differ significantly between identification methods (PERMANOVA based on Jaccard dissimilarities: *F* = 3.03, *R²* = 0.078, *P* = 0.500). However, multivariate dispersion differed significantly between methods (PERMDISP: *F* = 47.23, *P* < 0.001), indicating differences in the variability of prey community composition recovered among frogs.

The distribution of the 523 prey observations among taxonomic families also differed between methods (Table S5). Formicidae showed the largest discrepancy, with 200 observations recorded by metabarcoding compared with 34 by morphological identification. Other families were represented by similar numbers of observations between methods, including Staphylinidae (13 vs. 12 observations), Galumnidae (10 vs. 9), and Nothridae (8 vs. 8). Metabarcoding also recovered numerous observations assigned to fly, beetle, and springtail families that were rare or absent in the morphological dataset, whereas several mite groups, including Mesostigmata (e.g., Uropodidae) and Oribatida (e.g., Pelopidae and Oppiidae), were more strongly represented among morphological observations.

To assess whether prey size influenced metabarcoding detection, we compared the volumes of prey taxa detected and undetected by metabarcoding among morphologically identified prey. Taxa detected by metabarcoding were significantly larger than those recovered only through morphological identification (Wilcoxon rank-sum test: *P* < 0.001; effect size *r* = 0.300). Median prey volume was 0.119 mm^3^ for taxa detected by metabarcoding and 0.030 mm^3^ for taxa not detected by metabarcoding (Table S6), corresponding to a median difference of 0.088 mm^3^ (95% bootstrap CI = 0.005–0.155).

### Drivers of Alkaloid and Diet Variation

Frog body size (Table S1) ranged from 23.15 to 32.81 mm in snout–vent length (SVL) and from 6.90 to 9.79 mm in head width (HW). However, neither metric significantly explained variation in alkaloid profiles (PERMANOVA: SVL, *R²* = 0.049, *F* = 1.04, *P* = 0.404; HW *R²* = 0.055, *F* = 1.17, *P* = 0.284 for Bray–Curtis dissimilarities; SVL, *R²* = 0.052, *F* = 1.11, *P* = 0.309; HW, *R*² = 0.060, *F* = 1.29, *P* = 0.155 for Jaccard dissimilarities). Likewise, neither SVL nor HW significantly explained variation in prey composition when permutations were restricted within sampling events (PERMANOVA: SVL, *R²* = 0.048, *F* = 1.01, *P* = 0.494; HW, *R²* = 0.045, *F* = 0.95, *P* = 0.747).

We did not find any relationship between dissimilarity matrices of prey and alkaloid profiles. Mantel tests revealed no significant correlation between prey composition and alkaloid profiles, both at the individual compound level (*r* = 0.079, *P* = 0.282) and at the structural class level (*r* = -0.001, *P* = 0.457).

### Candidate Alkaloid-Source Associations

Using literature-derived alkaloid-arthropod associations in combination with individual-level diet data, 17 of the 130 detected alkaloids showed co-occurrence with prey taxa previously reported as known or putative sources of those compounds (Table S7).

**Fig. 5:**
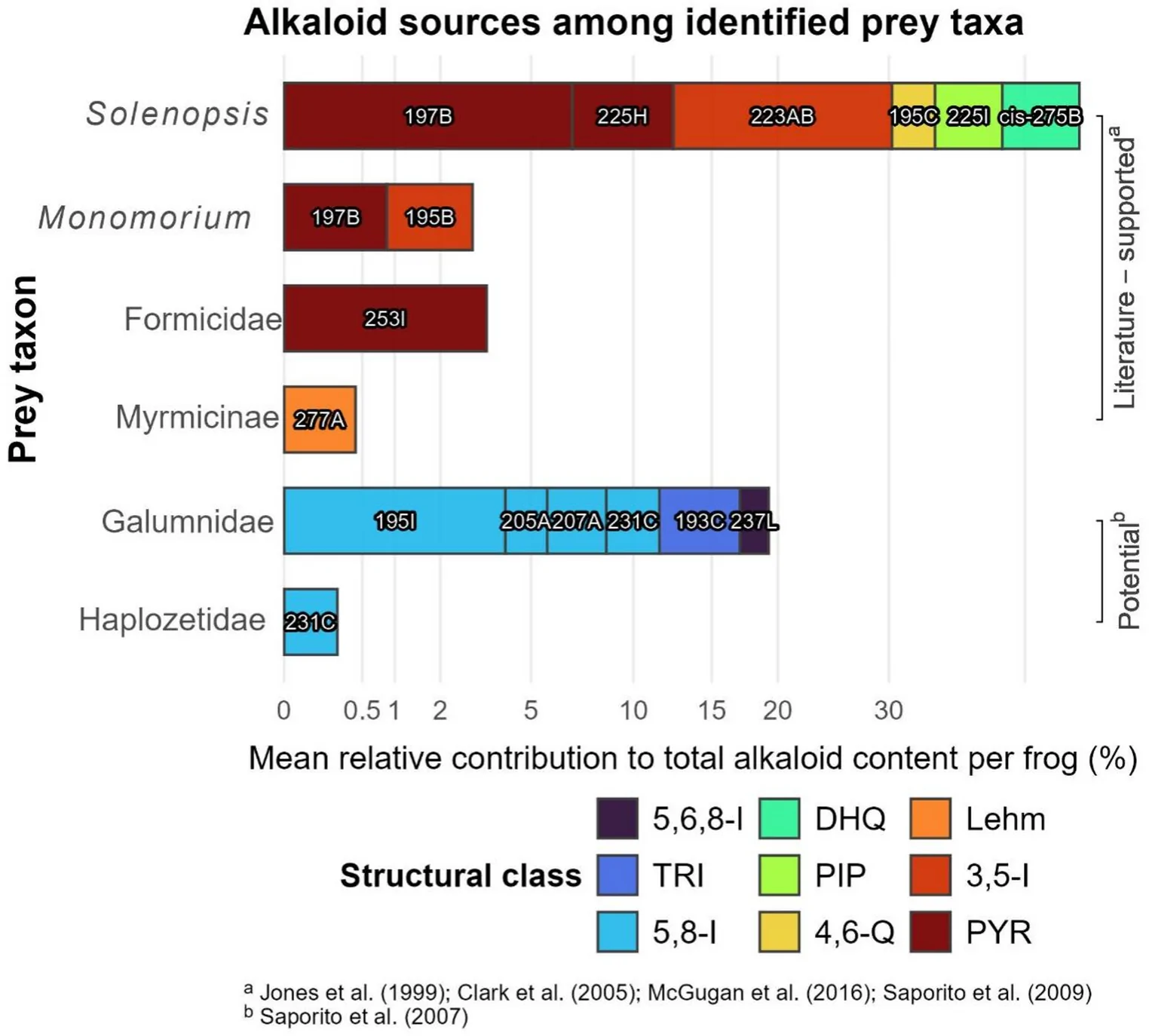
Literature-based assignment of alkaloids to candidate arthropod sources detected in the diet of *O. sylvatica*; stacked bar plots show the alkaloids for which a potential dietary source could be assigned based on compound-level matches and individual-level co-occurrence with prey taxa identified in stomach contents; bar lengths represent the mean relative contribution of each alkaloid to total alkaloid content per individual, displayed on a square-root-transformed scale; individual alkaloids are labelled within segments and colours indicate alkaloid structural classes; associations are classified as ‘literature-supported’ or ‘potential’ based on the strength of evidence from the literature; arthropod taxa are shown at genus or family level according to the taxonomic resolution of prey identification

These associations were dominated by ants (Formicidae), particularly the genus *Solenopsis*, whereas oribatid mites (Oribatida) accounted for a smaller number of matches. All ant-related associations were classified as ‘literature-supported’ based on direct literature evidence linking specific alkaloids to identified prey taxa. In contrast, evidence for oribatid mites was limited to a smaller number of studies and derived from mixed-family samples, resulting in ambiguous source attribution; these associations were therefore classified as ‘potential’ (Fig. 5).

Several ant-associated alkaloids were consistently detected across a large proportion of individuals. In particular, **225I** (PIP) and **253I** (PYR) co-occurred with Formicidae in 20 individuals, while **197B** (PYR) and **223AB** (3,5-I) co-occurred with *Solenopsis* in 18 and 17 individuals, respectively. Additional compounds, such as **225 H** (PYR) and **195I** (5,8-I) were detected in 14 individuals, although the latter was associated with oribatid mites.

Ant-derived alkaloids spanned multiple structural classes, including pyrrolidines (PYR), disubstituted indolizidines (3,5-I), decahydroquinolines (DHQ), disubstituted quinolizidines (4,6-Q), piperidines (PIP), and lehmizidines (Lehm). While some compounds were linked to multiple ant taxa, *Solenopsis* accounted for the majority of high-frequency matches, whereas *Monomorium* was only associated with comparatively few compounds and individuals. The lehmizidine **277 A** was associated with Myrmicinae at subfamily level.

In some cases, individual alkaloids have been reported from multiple arthropod sources. For example, pyrrolidine **253I** has been documented in both oribatid mites (Scheloribatidae) and ants (Formicidae) in the literature; in our data, this compound co-occurred with Formicidae in frog stomach contents.

In contrast, mite-associated alkaloids were fewer and less certain, involving two oribatid families. These included mostly Galumnidae, which were associated with several 5,8-indolizidines (**195I**, **205 A**, **207 A**, **231 C**), as well as a tricyclic alkaloid (**193 C**) and a 5,6,8-trisubstituted indolizidine (**237 L**). One compound (**231 C**) was linked to two oribatid families (Galumnidae and Haplozetidae).

Assigned compounds were limited in number and contributed modestly to total alkaloid content, with **197B** and **223AB** accounting for 6.85% and 4.03% per individual, respectively, and **253I** contributing 3.37%.

The majority of alkaloids showed no detectable co-occurrence with candidate source taxa within individual frogs.

## Discussion

### Broad Realized Diet and Alkaloid Diversity

Our first two objectives were to characterise individual-level alkaloid and prey richness and abundance, and our obtained results strongly supported our predictions. We found exceptionally high inter-individual variation in both prey composition and alkaloid profiles within a single *O. sylvatica* population. Such high fine-scale chemical diversity is consistent with results reported for other dendrobatids (Hovey et al. 2018; Basham et al. 2021; Albuquerque-Pinna et al. 2024; Porras-Brenes et al. 2025), and for *O. sylvatica*, specifically (McGugan et al. 2016).

Coverage analyses indicated that our sampling effectively captured the core alkaloid defence phenotype of the population. Although Chao2 extrapolation (Chao et al. 2014) suggested that ca. 22 rare alkaloids likely went undetected, Shannon and Simpson completeness demonstrated that the dominant, abundance-weighted portion of the chemical profile was well represented. This is biologically expected: poison frogs typically share a set of dominant alkaloids derived from common prey, whereas rare compounds arise from infrequent or stochastic dietary events (Stuckert et al. 2014; Lawrence et al. 2019; Sague et al. 2024).

In our system, the most common and potentially alkaloid-producing prey taxa were primarily ants in the genus *Solenopsis* and galumnid mites. We also observed a high abundance and richness of HTX and DHQ alkaloids in our samples, compounds that have so far been reported exclusively from myrmicine ants (Jones et al. 2012; Saporito et al. 2012). This finding further supports the hypothesis that ant consumption, particularly of taxa belonging to the Solenopsidini tribe, substantially contributes to the dominant alkaloid profile of this population of *O. sylvatica*.

Moreover, total alkaloid quantity increased significantly with alkaloid richness, indicating that frogs with more diverse alkaloid profiles accumulated greater quantities of toxins overall, rather than simply carrying more compounds at low abundance. Similar relationships have been reported in other poison frogs, where individuals consuming a wider diversity of alkaloid-bearing prey accumulate both more alkaloid types and a greater total quantity (Daly et al. 2005; Saporito et al. 2009; Santos et al. 2016; McGugan et al. 2016). However, whether this pattern reflects a generalist toxin acquisition strategy cannot be determined from our data.

Diet data also revealed substantial inter-individual variation, with frogs consuming diverse prey assemblages spanning multiple arthropod groups. Although ants and mites dominated prey richness, flies, beetles, and other taxa were also frequently detected, suggesting that *O. sylvatica* in this regenerating cacao system exploits a broader dietary niche than is typically assumed for chemically defended dendrobatids, which are often characterized as specialists on ants and mites (Toft 1981; Caldwell 1996; Darst et al. 2005). Still, several taxa were consistently detected across individuals, including *Archegozetes* sp., *Solenopsis terricola*, *Pheidole zeteki*, and *Pheidole flavens*. The high abundance of these arthropod taxa in a broader leaf-litter community study conducted at the same site further indicates that they were locally abundant in the environment (Tartara et al. 2026). Notably, several of these recurrent prey taxa belong to groups commonly associated with alkaloid sequestration in poison frogs (Daly et al. 1994; Saporito et al. 2009, 2012). In particular, ant genera frequently consumed in our study, including *Solenopsis*, *Pheidole*, *Wasmannia*, and *Octostruma*, have previously been reported from alkaloid-containing poison frog diets or suggested as candidate alkaloid sources (Fox and Adams 2022; Jones et al. 2012; McGugan et al. 2016). Likewise, the repeated detection of galumnid mites is notable given the recognized role of oribatid mites in poison frog chemical ecology (Saporito et al. 2007a, b, 2011, 2012, 2015; Heethoff et al. 2016).

In conclusion, our results point less toward strict dietary specialization and more toward a broad realized diet (Martin et al. 2025). Such dietary breadth may be particularly advantageous in regenerating agroforestry habitats, where arthropod communities can vary substantially across space and time (Tartara et al. 2026). Under these conditions, frogs may consume alkaloid-associated prey alongside a wider range of available arthropod resources. However, without timely data on prey availability, it remains unresolved whether this broad dietary breadth reflects non-random feeding within a broad trophic niche or variation in the arthropod community.

### Metabarcoding and Morphological Detection Performance in Poison Frog Trophic Systems

Our second objective was to evaluate the relative performance of metabarcoding and morphological identification for reconstructing *O. sylvatica* diet. As expected, the two approaches recovered partially distinct prey spectra, reflecting their different methodological strengths and limitations.

Morphological identification recovered fewer taxa overall, but consistently detected several groups of small, sclerotized arthropods, particularly mites, that were comparatively less represented in the metabarcoding dataset. In contrast, metabarcoding recovered substantially higher prey richness and a broader taxonomic spectrum than morphological identification, particularly through the detection of numerous fly, ant, and beetle taxa. Similar increases in prey recovery and taxonomic resolution have been reported in other metabarcoding-based diet studies, especially for highly digested and morphologically unidentifiable prey remains (Clare 2014; Egeter et al. 2015; Kopp et al. 2023). These patterns likely reflect how the two methods interact with the digestion process rather than differences in intrinsic methodological accuracy.

Biases toward persistent hard-bodied prey have long been recognized in stomach content analyses based on visual identification (Kress et al. 2015; Sakaguchi et al. 2017), which probably contributed to the strong representation of several mite groups in the morphological dataset, particularly Mesostigmata, Pelopidae, Oppiidae, and Uropodidae. Metabarcoding depends instead on the persistence of amplifiable DNA (Holman et al. 2022), therefore smaller prey contains less tissue and may therefore lose detectable DNA more rapidly during digestion. However, our results do not necessarily suggest that metabarcoding inherently performs poorly for small or sclerotized taxa. Several groups, including Galumnidae, Trhypochthoniidae, Nothridae, and Staphylinidae, were still recovered at comparatively similar richness by both methods. This is consistent with previous studies showing that, when DNA preservation and amplification are adequate, metabarcoding can successfully recover very small arthropods (Deagle et al. 2019; Elbrecht and Leese 2015).

Successful metabarcoding depends on the availability of matching reference sequences (Porter et al. 2019; Basset et al. 2022). Many tropical arthropod groups, particularly mites and other potential alkaloid-producing taxa, remain poorly represented in molecular libraries, limiting taxonomic assignment even when sequences are successfully recovered (Amundsen and Sánchez-Hernández 2019). In our dataset, this likely contributed to the comparatively finer taxonomic resolution achieved through morphological identification for several mite groups. In contrast to morphology, metabarcoding also requires destructive processing of stomach contents prior to DNA extraction, preventing unresolved sequences from being subsequently re-examined morphologically.

Finally, our results show that morphology and metabarcoding provide complementary rather than interchangeable perspectives on poison frog diets. Neither method alone fully captured the diversity of prey consumed by *O. sylvatica*, and, based on the evidence from our system, combining both provided the most complete reconstruction of chemically relevant diets and potential alkaloid sources.

For future studies, separating stomach contents into different prey-size fractions prior to DNA extraction and metabarcoding could help reduce amplification bias, particularly in prey-rich stomach samples where DNA from larger prey may dominate and mask smaller taxa. At the same time, improving tropical molecular reference databases still remains essential, as improved taxonomic coverage may eventually allow metabarcoding alone to recover chemically relevant prey diversity at sufficient resolution for diet-alkaloid studies.

### Relationship Between Prey Composition and Alkaloid Profiles

Our third objective was to test whether similarity in diet composition corresponded to similarity in alkaloid profiles among individuals. Despite the pronounced variation observed in both prey composition and chemical defence, we did not detect a significant relationship between diet and alkaloid similarity across frogs.

One likely explanation is the mismatch in temporal scale between the two datasets. Stomach contents represent only a short-term record of recently consumed prey, whereas alkaloids can accumulate and persist in poison frogs over much longer periods (Daly et al. 2005; Krieger et al. 2022; O’Connell et al. 2021). As a result, the prey detected at the time of sampling may not necessarily reflect the prey responsible for the alkaloid profile currently stored in the skin.

Another factor is that prey contributing strongly to alkaloid sequestration may occur only rarely in stomach samples. Poison frog alkaloid profiles are thought to depend on a relatively small subset of chemically relevant arthropods (Daly et al. 1994; Saporito et al. 2012), and even infrequently consumed prey may contribute disproportionately to chemical defence. Such prey could easily be missed in short-term dietary snapshots, particularly in systems with highly diverse arthropod communities such as the Ecuadorian Chocó.

Limited knowledge of alkaloid occurrence in tropical arthropods further complicates these interpretations. Although some ants and oribatid mites are widely recognized as important alkaloid sources (Daly et al. 1994; Jones et al. 2012; Saporito et al. 2007a, b, 2011, 2015; Heethoff et al. 2016), many of the prey taxa recovered in our study have never been chemically characterized.

Our literature-based co-occurrence analysis further highlighted this gap. Although several alkaloid classes detected in *O. sylvatica* could be broadly associated with ants and oribatid mites, only a small subset of prey taxa recovered from stomach contents could be matched to previously reported alkaloid-producing arthropods. Two frequently consumed ant genera, *Solenopsis* and *Monomorium*, co-occurred with alkaloid classes that have previously been associated with these taxa, particularly PYR and 3,5-I alkaloids (Jones et al. 2012; Saporito et al. 2012). In other cases, prey belonged to taxonomic groups known to contain alkaloid-bearing arthropods, but without species-specific chemical data it remains impossible to determine whether the consumed taxa themselves contributed to alkaloid sequestration. This was particularly evident for oribatid mites, where taxonomic diversity in our samples was high but chemical information remains extremely scarce. For example, galumnid mites (Oribatida) were repeatedly detected in frogs containing alkaloids previously linked to oribatid mites, including TRI, 5,6,8-I, and 5,8-I alkaloids. However, these associations remain indirect (‘potential’) because alkaloid presence can vary strongly even among closely related arthropod taxa (Heethoff et al. 2016).

Our results therefore highlight how little is still known about alkaloid distribution across tropical arthropod communities, and how strongly this limits fine-scale reconstruction of diet-alkaloid relationships in poison frogs.

## Conclusion

Our study highlights the remarkable trophic and chemical heterogeneity that can exist within a single population of *Oophaga sylvatica* inhabiting a regenerating tropical system. Rather than relying on a narrow set of prey, frogs consumed a broad diversity of arthropods, including prey groups commonly associated with alkaloid sequestration. These patterns suggest that chemical defence in this population occurs within a broader and flexible trophic niche, although the extent to which particular prey are preferentially consumed cannot be determined without information on prey availability.

At the same time, our results emphasize how difficult it remains to directly connect prey consumption with alkaloid profiles in wild poison frogs. The absence of a relationship between recent diet and chemical similarity among individuals likely reflects the cumulative nature of alkaloid sequestration, together with the still limited understanding of which tropical arthropods actually possess alkaloids. Our literature-based source assignment clearly showed that only a small fraction of prey taxa could currently be linked to known alkaloid-bearing groups, despite the high prey diversity recovered from stomach contents.

Methodologically, we show that metabarcoding and morphological identification recovered complementary components of the diets, highlighting the importance of integrative approaches when studying chemically defended trophic systems under the current state of knowledge.

Our findings show that diet-alkaloid relationships emerge from the interaction of ecological dynamics, alkaloid accumulation processes, and methodological limitations operating across different temporal scales. Expanding the taxonomic and chemical characterization of tropical arthropod communities is therefore essential for resolving the ecological mechanisms underlying poison frog chemical defence.

## Supplementary Information

Below is the link to the electronic supplementary material.

Supplementary Material 1

## Data Availability

The data that support the findings of this study is available on Figshare at https://doi.org/10.6084/m9.figshare.32743443.
