# Supplementary material for: Disentangling Chemical Defence and Prey Composition of Oophaga sylvatica
Source: J Chem Ecol. 2026 Nov 10;52(6):102. doi: 10.1007/s10886-026-01780-w (PMC13751695; doi:10.1007/s10886-026-01780-w)
Supplement: Supplementary file 1 [file 10886_2026_1780_MOESM1_ESM.docx]

**Disentangling chemical defence and prey composition of *Oophaga sylvatica***

*Arianna Tartara^1,6^, Karla Neira-Salamea^2,3,4^, Alexander Keller^5^, Mark-Oliver Rödel^2^, Florian Frenzel^1,6^, Michael Heethoff^1^ and Ralph A. Saporito^7^*

**^1^**Animal Evolutionary Ecology, Ecological Networks, Technical University of Darmstadt, Darmstadt, Germany

**^2^**Museum für Naturkunde, Leibniz Institute for Evolution and Biodiversity Science, Berlin, Germany

**^3^**Humboldt-Universität zu Berlin, Faculty of Life Sciences, Berlin, Germany

**^4^**Grupo de Investigación en Ecología y Evolución en los Trópicos-EETrop, Universidad de Las Américas, Quito, Ecuador

^5^Cellular and Organismic Networks, Faculty of Biology, Ludwig-Maximilian-Universität, Faculty of Biology, Münich, Germany

^6^Institute of Soil Science & Land Evaluation, Department of Soil Biology, University of Hohenheim, Stuttgart, Germany

^7^John Carroll University, Department of Biology, University Heights, Ohio, USA

**Supplementary information**

**Index**

Table S1…………………………………………………………………….............. Body traits and sampling date

Fig. S1………………………………………………………………………………………….... Sequencing depth

Fig. S2……………………………………………………………….. Enrichment distribution in negative controls

Fig. S3……………………………………………………………….. Taxonomic composition in positive controls

Fig. S4…………………………………………………………………... Distribution of mean alkaloid abundance

Table S2…………………………………………………………………………….Diet data integration procedure

Table S3…………………………………………………………………………………….. Unidentified alkaloids

Fig. S5……………………………………………………………………..... Rarefaction and extrapolation curves

Table S4………………………………………………………………….………………….. Sample completeness

Fig. S6……………………………………………………………………………………………....... Linear model

Fig. S7……………………………………………………………………………………… Structural class profile

Fig. S8………………………………………………………………………………………...…………Diet profile

Table S5………………………………………………………………………… Prey families detected by method

Table S6…………………………………………………………………………………. Summary of prey volume

Table S7………………………………………………………………………….. Alkaloid-arthropod associations

**Fig. S1.** Sequencing depth (total reads) in negative controls and biological samples

**Fig. S2.** Log2 enrichment of taxa detected in negative controls relative to biological samples; the grey dotted line indicates the empirical bulk mean estimated by iterative trimming, representing the expected aerosol-contamination baseline; taxa exceeding the outlier threshold (bulk mean + 2 SD; red dashed line) are considered anomalously enriched beyond within project aerosol-contamination levels and thus considered as external lab contamination

**Fig. S3.** Relative abundance of taxa detected in positive controls (*Bombus terrestris* standard); the horizontal dashed line indicates the 0.1% threshold applied to remove low-abundance detections attributed to index-jumping and cross-contamination between project samples

**Table S1.** Body traits and sampling date for the 23 *O. sylvatica* specimen. Body measurements’ abbreviations stand for: snout-vent length (SVL) and head width (HW). KN stands for Karla Neira and AT stands for Arianna Tartara, both authors of this paper. All body measurements are reported in mm

| **Sample** | **Date** | **Collector** | **SVL** | **HW** |
| --- | --- | --- | --- | --- |
| CR13_2204_22_1 | 22/04/2022 | KN-AT | 27.81 | 9.38 |
| CR13_2204_22_2 | 22/04/2022 | KN-AT | 32.27 | 9.00 |
| CR13_0807_22_1 | 08/07/2022 | KN-AT | 32.79 | 9.78 |
| CR13_0807_22_2 | 08/07/2022 | KN-AT | 30.76 | 8.25 |
| CR13_1708_22_1 | 17/08/2022 | KN | 32.40 | 8.99 |
| CR13_1708_22_3 | 17/08/2022 | KN | 28.53 | 7.10 |
| CR13_1708_22_4 | 17/08/2022 | KN | 32.81 | 9.49 |
| CR13_2010_22_4 | 20/10/2022 | KN | 31.90 | 9.13 |
| CR13_2010_22_5 | 20/10/2022 | KN | 23.19 | 7.83 |
| CR13_1403_23_1 | 14/03/2023 | KN | NA | NA |
| CR13_1403_23_2 | 14/03/2023 | KN | 31.91 | 8.79 |
| CR13_1403_23_3 | 14/03/2023 | KN | 27.34 | 9.05 |
| CR13_1403_23_4 | 14/03/2023 | KN | 23.15 | 6.90 |
| CR13_1403_23_5 | 14/03/2023 | KN | 31.61 | 9.10 |
| CR13_1805_23_1 | 18/05/2023 | KN | 28.25 | 7.87 |
| CR13_1805_23_2 | 18/05/2023 | KN | 30.78 | 9.52 |
| CR13_1805_23_3 | 18/05/2023 | KN | 28.38 | 8.44 |
| CR13_1805_23_4 | 18/05/2023 | KN | 29.86 | 9.16 |
| CR13_1805_23_5 | 18/05/2023 | KN | 32.50 | 9.30 |
| CR13_1805_23_6 | 18/05/2023 | KN | 30.79 | 9.20 |
| CR13_1805_23_7 | 18/05/2023 | KN | 31.10 | 9.79 |
| CR13_1805_23_8 | 18/05/2023 | KN | 30.61 | 9.43 |
| CR13_1805_23_9 | 18/05/2023 | KN | 29.20 | 8.60 |

**Fig. S4.** Distribution of mean alkaloid abundance across all detected alkaloids in *O. sylvatica*

**Table S2.** Decision workflow for integrating morphological and metabarcoding prey identifications

| Step | Comparison | Criterion | Outcome |
| --- | --- | --- | --- |
| 1 | Species level | Order, family, genus, and species agree between methods | One concordant species-level record |
| 2 | Genus level | Among records not matched at species level: order, family, and genus agree | One concordant genus-level record |
| 3 | Family level | Among records not matched at species or genus level: order and family agree | One concordant family-level record |
| 4 | No concordance | No match at species, genus, or family level | Records retained as method-specific |
| 5 | Duplicate records | Same frog and same order–family–genus–species combination | Collapsed to a single record |
| 6 | Final integration | Concordant records | Single record retained using metabarcoding taxonomy; method-specific records retained separately |

**Table S3.** The retention times and molecular weight for putatively new alkaloids that could not be matched to previously published mass spectral data

| **Alkaloid Class** | **Retention Time** | **Molecular Weight** | **No. Frogs** |
| --- | --- | --- | --- |
| Unclass | 7.66 | 207 | 7 |
| Unclass | 7.78 | 207 | 2 |
| Unclass | 10.93 | 213 | 5 |
| Dehydro-5,8-I | 12.03 | 235 | 1 |
| 5,8-I | 10.21 | 237 | 18 |
| Unclass | 13.96 | 269 | 3 |
| Unclass | 15.78 | 269 | 2 |
| Unclass | 14.59 | 273 | 2 |
| HTX | 15.99 | 287 | 16 |
| Unclass | 17.15 | 299 | 1 |

**Fig. S5** Rarefaction (solid line) and extrapolation (dashed line) curves of alkaloid richness based on Hill numbers of order q = 0. Panel a) shows the approach based on sample size (N) and panel b) shows the coverage-based approach, representing sample completeness

**Table S4.** Observed, estimated and sample coverage (completeness) for alkaloid richness, Shannon diversity, and Simpson diversity

|  | **Observed** | **Estimated ± SE** | **95% Lower CI** | **95% Upper CI** | **Completeness** |
| --- | --- | --- | --- | --- | --- |
| **Alkaloid richness** | 130 | 151.6 ± 12.0 | 128.1 | 175.0 | 0.86 |
| **Shannon diversity** | 91.6 | 97.3 ± 2.3 | 92.9 | 101.7 | 0.94 |
| **Simpson diversity** | 76.9 | 79.7 ± 1.9 | 76.0 | 83.4 | 0.97 |

**Fig. S6** Linear regression of total alkaloid quantity as a function of alkaloid richness for individual frogs; the fitted regression equation was total alkaloid quantity = −31.5 + 2.8 × alkaloid richness and the respective *R^2^* is reported; residual normality was supported by a Shapiro–Wilk test (*W* = 0.921, P = 0.069) and visual inspection of residual plot

**Fig. S7** Panel a) shows alkaloids richness per class, and panel b) shows total alkaloid quantity (abundance) per class across the sampled *O. sylvatica*

**Figure S8.** Prey composition across the 23 individuals of *O. sylvatica* by broader taxonomic groups

**Table S5.** Taxa detected by metabarcoding and morphological identification, ranked separately by the number of observations detected by each method; N indicates the number of prey observations assigned to each taxon

| Rank | Metabarcoding | N_Metabarcoding | Morphology | N_Morphology |
| --- | --- | --- | --- | --- |
| 1 | Formicidae | 200 | Formicidae | 34 |
| 2 | Ceratopogonidae | 65 | Staphylinidae | 12 |
| 3 | Trhypochthoniidae | 17 | Galumnidae | 9 |
| 4 | Staphylinidae | 13 | Trhypochthoniidae | 9 |
| 5 | Galumnidae | 10 | Nothridae | 8 |
| 6 | Chironomidae | 8 | Parasitidae | 8 |
| 7 | Coleoptera spp. | 8 | Mesostigmata spp. | 6 |
| 8 | Diptera spp. | 8 | Galumnidae | 5 |
| 9 | Nothridae | 8 | Collembola spp. | 4 |
| 10 | Paronellidae | 7 | Parasitidae | 4 |
| 11 | Chrysomelidae | 6 | Pelopidae | 4 |
| 12 | Entomobryidae | 6 | Formicidae | 3 |
| 13 | Ascidae | 5 | Oppiidae | 3 |
| 14 | Curculionidae | 5 | Oribatida spp. | 3 |
| 15 | Parasitidae | 5 | Staphylinidae | 3 |
| 16 | Brachystomellidae | 4 | Arachnida spp. | 2 |
| 17 | Cantharidae | 4 | Nothridae | 2 |
| 18 | Carabidae | 4 | Uropodidae | 2 |
| 19 | Lampyridae | 4 | Araneae spp. | 1 |
| 20 | Psychodidae | 4 | Chthoniidae | 1 |
| 21 | Sciaridae | 4 | Dampfiellidae | 1 |
| 22 | Arachnida spp. | 3 | Euphthiracaridae | 1 |
| 23 | Araneidae | 3 | Eviphidoidea | 1 |
| 24 | Cecidomyiidae | 3 | Gasteropoda f | 1 |
| 25 | Leiodidae | 3 | Haplozetidae | 1 |
| 26 | Tachinidae | 3 | Isopoda f | 1 |
| 27 | Chromadorea spp. | 2 | Lohmannidae | 1 |
| 28 | Erebidae | 2 | Prostigmata f | 1 |
| 29 | Erythraeidae | 2 | Rhynchoribatidae | 1 |
| 30 | Figitidae | 2 | Schizopteridae | 1 |
| 31 | Hymenoptera spp. | 2 | Sternorrhyncha f | 1 |
| 32 | Lauxaniidae | 2 | NA | NA |
| 33 | Muscidae | 2 | NA | NA |
| 34 | Phthiracaridae | 2 | NA | NA |
| 35 | Podocinidae | 2 | NA | NA |
| 36 | Scelionidae | 2 | NA | NA |
| 37 | Schizopteridae | 2 | NA | NA |
| 38 | Stratiomyidae | 2 | NA | NA |
| 39 | Trematuridae | 2 | NA | NA |
| 40 | Achipteriidae | 1 | NA | NA |
| 41 | Anamorphidae | 1 | NA | NA |
| 42 | Anyphaenidae | 1 | NA | NA |
| 43 | Apidae | 1 | NA | NA |
| 44 | Araneae spp. | 1 | NA | NA |
| 45 | Archipsocidae | 1 | NA | NA |
| 46 | Blattisociidae | 1 | NA | NA |
| 47 | Braconidae | 1 | NA | NA |
| 48 | Clambidae | 1 | NA | NA |
| 49 | Cleridae | 1 | NA | NA |
| 50 | Collembola spp. | 1 | NA | NA |
| 51 | Culicidae | 1 | NA | NA |
| 52 | Drosophilidae | 1 | NA | NA |
| 53 | Ephydridae | 1 | NA | NA |
| 54 | Gryllidae | 1 | NA | NA |
| 55 | Hydrophilidae | 1 | NA | NA |
| 56 | Ichneumonidae | 1 | NA | NA |
| 57 | Laelapidae | 1 | NA | NA |
| 58 | Limoniidae | 1 | NA | NA |
| 59 | Lycidae | 1 | NA | NA |
| 60 | Micropterigidae | 1 | NA | NA |
| 61 | Noctuidae | 1 | NA | NA |
| 62 | Oribatida spp. | 1 | NA | NA |
| 63 | Pentatomidae | 1 | NA | NA |
| 64 | Phlaeothripidae | 1 | NA | NA |
| 65 | Phoridae | 1 | NA | NA |
| 66 | Polyxenidae | 1 | NA | NA |
| 67 | Psychidae | 1 | NA | NA |
| 68 | Ptiliidae | 1 | NA | NA |
| 69 | Scatopsidae | 1 | NA | NA |
| 70 | Syrphidae | 1 | NA | NA |
| 71 | Theridiidae | 1 | NA | NA |
| 72 | Trichogrammatidae | 1 | NA | NA |
| 73 | Trigonidiidae | 1 | NA | NA |

**Table S6.** Summary of prey volume (mean and median), count and metabarcoding detection success rate of prey items that were identified morphologically

| Metabarcoding detection | Prey volume - mean (mm^3^) | Prey volume - median (mm^3^) | No. prey items |
| --- | --- | --- | --- |
| No | 0.087 | 0.030 | 53 |
| Yes | 0.206 | 0.119 | 75 |

**Table S7.** Literature-based assignment of alkaloids detected in *O. sylvatica* to candidate dietary arthropod sources found in frog stomachs; alkaloids are listed with the matched prey taxon (genus, subfamily, or family level), alkaloid structural class, source type (‘literature-supported’ vs. ‘potential’), number of individuals showing alkaloid and alkaloid-producing prey co-occurrence, and mean relative contribution to total alkaloid content per individual (%); references indicate the literature sources of alkaloid-arthropod associations.

| Alkaloid | Taxon matched | Class | Source type | No. matched samples | Mean qty (% total) | References |
| --- | --- | --- | --- | --- | --- | --- |
| 193C | Galumnidae | TRI | Potential | 7 | 0.41 | Saporito et al., 2007 |
| 195B | *Monomorium* | 3,5-I | Literature-supported | 1 | 0.62 | Jones et al., 1999 |
| 195C | *Solenopsis* | 4,6-Q | Literature-supported | 1 | 0.08 | Jones et al., 1999 |
| 195I | Galumnidae | 5,8-I | Potential | 14 | 4.04 | Saporito et al., 2007 |
| 197B | *Monomorium* | PYR | Literature-supported | 1 | 0.87 | Jones et al., 1999 |
| 197B | *Solenopsis* | PYR | Literature-supported | 18 | 6.85 | Jones et al., 1999 |
| 205A | Galumnidae | 5,8-I | Potential | 9 | 0.13 | Saporito et al., 2007 |
| 207A | Galumnidae | 5,8-I | Potential | 10 | 0.29 | Saporito et al., 2007 |
| 223AB | *Solenopsis* | 3,5-I | Literature-supported | 17 | 4.03 | Jones et al., 1999 |
| 225H | *Solenopsis* | PYR | Literature-supported | 14 | 0.84 | Jones et al., 1999 |
| 225I | *Solenopsis* | PIP | Literature-supported | 20 | 0.31 | Jones et al., 1999 |
| 231C | Galumnidae | 5,8-I | Potential | 11 | 0.19 | Saporito et al., 2007 |
| 231C | Haplozetidae | 5,8-I | Potential | 11 | 0.19 | Saporito et al., 2007 |
| 237L | Galumnidae | 5,6,8-I | Potential | 1 | 0.03 | Saporito et al., 2007 |
| 253I | Formicidae | PYR | Literature-supported | 20 | 3.37 | Saporito et al., 2009 |
| 277A | Myrmicinae | Lehm | Literature-supported | 7 | 0.34 | McGugan et al., 2016 |
| *cis*-275B | *Solenopsis* | DHQ | Literature-supported | 1 | 0.44 | Jones et al., 1999 |
